# Spirit of the British and American Periodicals

**Published:** 1840-01-01

**Authors:** 


### Spirit of the British and American Periodicals, &c.

---

### SKETCHES OF MEDICINE IN THE EAST. By VALENTINE MOTT, M.D. OF NEW YORK.

These sketches, or as their able author terms them gleanings, are contained in the American Journal of the Medical Sciences for August, 1839. They present a good deal to interest us, and may help us to the formation of a just opinion on the probability of a political regeneration of the East.

1. *The Medical School of Egypt.*—Every body knows that Mahomet Ali's policy has been to surround himself with European agents of his despotism. Vanity has, no doubt, had something to do with this—selfishness has had much more. But, whatever the motive, the effects, in the instance of medicine and a medical establishment, are indubitably beneficial.

"From a fear too no doubt, that a sufficient inducement could not be held out, for foreigners of merit to take up their residence in this benighted country, the Pasha has from time to time been in the habit of sending to the medical schools of Europe, and particularly of France, a number of young Arabs, to be educated at the expense of the government. In this way a ready communication is had with the foreign practitioners and the native eléves of the country, who assemble in the hospitals and medical schools, until the former have acquired a sufficient knowledge of the Arabic language to impart instruction to them in their native tongue. In this way we have witnessed the lessons of the professor conveyed to the pupil by a young Egyptian physician who had been educated in Paris, French being the language used for this purpose. The Arabic, as I was informed by the professors, is extremely difficult to be acquired, and those only who had resided in the country for eight or ten years, were able to read it and above all to speak and understand it sufficiently well to hold intercourse with the natives and impart instruction directly to the pupils."

"The medical school of Egypt, which for some years has been located at *Abou-Zabel*, is now removed to *Esbekie*, in the immediate vicinity of Cairo; the former being too remote from the capital, to enable the professors, from their necessary duties in private practice, to do full justice to the institution. The school makes part of a large and well arranged military hospital, beautifully and pleasantly situated on the western bank of the Nile, in the suburbs of Cairo. This hospital contained thirteen hundred patients when we visited it. The immediate connection of the medical school with this large hospital, together, making one great edifice, is in my opinion an admirable arrangement for the benefit of the pupils, and well deserving of imitation in other and more enlightened countries. The lecture-rooms of the professors are all exceedingly well arranged, and the amphitheatre for anatomy is particularly well constructed, with an abundance of light from a cupola on the top. A large and well arranged pharmacy, with specimens of every kind of domestic and foreign drug, while it abundantly supplies medicines to the wants of the hospital, serves as a means of instructing the students. A large laboratory is connected with it, in which the new chemicals, such as alkaloids, and others, are prepared, to answer the demands of the physicians, and at the same time extend information to the pupils, by making them acquainted with chemical pharmacy.

The number of pupils attending the lectures at the time of our visit, was two hundred and sixty. They are not only attendants upon the lectures of the professors, but residents in the hospital, in order to observe the treatment of the patients, and to become familiar with the almost endless forms and features of disease.

They are all educated at the public expense, have their quarters in the hospital, where they eat and sleep, and are obedient to a regular military and medical discipline, and rank as *sous aides*, in the surgical staff of the army. Here they are compelled to remain from three to four years in the constant pursuit of their studies, and in the regular observance of disease, at all times obedient to the call of their superiors, and ready to administer to the wants of the patients.

The beautiful order and methodical arrangements, as well as neatness, in every part of this establishment, surprised and delighted me. It unites the activity of the French, with the cleanliness and good system of the German hospitals, and therefore may be said to have the excellence of both."

The anatomical museum is very respectable, and will serve as the nucleus of a good collection. It consists mostly of bones, casts, and wax models, with the excellent tributary aids, of parts, and the whole subject, of the ingenious invention of Doctor Auzoux. From the expense of alcohol, and the great waste, owing to the excessive heat and dryness of the climate of Egypt, few or no specimens of morbid parts can be preserved as wet preparations. They are compelled to resort to drawings and wax models, to perpetuate their similitude.

The apparatus for the illustration of the physical sciences is neat, and sufficiently ample.

The Civil Hospital is situated in the city of Cairo, and is located in a spacious building, but recently one of the palaces of Mahomet Ali. It is placed very favourably for good air, near the principal square of this very curious and truly oriental city. It is an admirable transfer of the noble and superfluous domain of a single individual to humane and charitable purposes, to the wants, necessities, and the afflictions of the poor and the diseased. Dr. Mott was informed that it had only been established for one year, and was but the beginning of an asylum and a home for the suffering and the sick.

"It contained between two and three hundred patients, besides apartments, especially appropriated for a lying-in establishment. Although there is a male and female department in the same building, there is the peculiar eastern vigilance, and harem-like care, that the females shall not even be seen by the male patients. On no pretence, whatever, is any male admitted into the female part of the hospital unless he be a professional man, and then he must accompany a medical officer of the establishment, who only has authority to introduce him.

Connected with this maternité, is a school for the education of young women, to fit them properly to be accoucheuses or sages femmes. It has a well organised class of young females from the age of fifteen to twenty, under the care of a French professor, aided by a young Arab, whose acquaintance with the French language enabled the pupils to comprehend readily the lessons of the principal. The class consisted, on the day of our visit, of sixteen. They were dressed as Europeans, were very neat and respectable in their appearance, and exhibited various tints, and shades of colour, from the tawny Arab, to the jet black Nubian and Abyssinian.

They were all assembled in the class, at their lessons, when we entered, and were receiving instruction from the professor. Their note-books were in Arabic and French. I was requested to test the practical knowledge of one of them on the mannikin. One, the most convenient, and as black as ebony, was requested to come forward. Different questions in French were put through the young Egyptian, and on the machines the pupil proved by her manipulations with the fœtus, that she not only comprehended perfectly the question, but that she understood well the subject.

When their knowledge is thought sufficient, they are permitted to exercise the art upon the patients of the institution. In this way, after a residence of some time in the hospital, subjected to regular discipline and instruction, they become very competent practitioners of this branch of the profession. They informed me that all of them were educated at the expense of the Pasha, and that his object was to place them in the harems, and thereby dispense with male obstetricians; that Mahomet Ali, from time to time, was in the practice of purchasing young females at the slave market at Cairo, and placing them in the maternité for instruction. In this way he kept up a constant supply for the wants of the different harems of his family and favourites.

This establishment is undoubtedly founded upon the liberal and humane plans of the French, who annually educate, and send forth a large number of well instructed and competent young women, not only in every direction through their own provinces, but into other countries. It is to be hoped, that in Egypt, a more enlarged and liberal view will be taken of this system, and ere long that its salutary and benign influence, will be extended, far beyond the gardens and walls of the harems; and that the almost countless poor, may receive something in return for what they labour so hard to support.

Every facility seemed to be afforded in this obstetric school, in preparations, apparatus and instruments, as well as the living subject, to make the pupils competent and useful practitioners."

2. *Medical School at Athens.*—"An attempt is making at Athens, the present capital of the modern Greek empire, to organize a medical school, by several well educated and respectable Bavarian physicians and surgeons, who are attached to the court, and whom King Otho has induced to settle in his country. At the time of my visit, (April 1838), they had from nine to twelve pupils, natives of the country, who were lectured to by the Germans in the modern Greek language; the professors from a residence of several years in the country, being able to impart instruction to the pupils in their native tongue. Although it is the merest beginning of a medical school, it is nevertheless praiseworthy and honourable to its present founders, and may be the germ of an institution, which shall move onward hand in hand, with the regeneration of poor fallen Greece."

Athens possesses at this moment a most excellent military hospital, arranged upon the modern European plan, capable of containing very comfortably several hundred patients. Also a highly respectable and well arranged cabinet of Natural History.

"From what I saw of the diseases of Greece, both in the hospital at Athens, and in the practice of two of the physicians attached to the court of King Otho, they appear to be generally of an inflammatory character. This is especially true with those of a febrile form, and the brain seems to be the organ in which this is most particularly manifested. All their endemic fevers commence with strong synochal symptoms, and are remarkably fatal to new comers during the cerebral determination. If they escape from this stage, they are precipitated very speedily into a profound typhus, or it assumes an intermittent fever of the quotidian or tertian types. These latter continue with great obstinacy in defiance of quinine and arsenic, and frequently terminate fatally in general hydropic effusions, and hypertrophy of the spleen.

The Greeks are generally a very temperate people, and from living in a mild climate, subject to few sudden variations, and an atmosphere the purest and possessing a clearness and transparency that far exceeds any thing I ever met with in any other country, are consequently a hardy and vigorous race. The great distance at which an object can be seen, and the deception thence arising in the computation of hours or miles in travelling, was a frequent remark with my companions and myself, and we found that the same thing had been noticed by foreigners resident in the country.

It ought to be recollected by all who visit Greece, that an abstemious mode of living, and particularly great moderation in the use of stimulating drinks, is essential to the preservation of health. From my own observation less wine can be borne in that climate, than in any I ever visited. Every year at Athens I found that the endemic fever was fatal to many Europeans, from imprudence in indulging in their accustomed quantity of stimulating drinks, particularly wines."

3. *The Lepra Græcorum.*—" The Lepra of Greece I had long felt a great desire to examine, and took the opportunity of visiting some cases of it through the politeness of Dr. R. and of making numerous inquiries of him as to the general character and treatment of this ancient and curious disease. It bears a very striking resemblance to the venereal disease, in its primary and secondary or confirmed stages. So marked indeed is the coincidence, that it does not appear to me to be going too far to say that it may be the parent or progenitor of lues venerea. It attacks primarily the genital organs, then the throat, skin, and lastly the bones. It is generally a more chronic affection than lues as we commonly see it, but it assails the same parts and resembles many cases of lues, such as I have often seen; so much so, that Dr. Jackson of New York, my travelling companion, as well as myself, confessed, that it would not be in our power to discriminate between them. The lepra on the genitals, and in the throat is commonly attended with more hardened and elevated ulcerations, with more hypertrophy of the surrounding tissues, than ordinary cases of lues, but such precisely, as I have seen in the venereal disease. The affection of the skin resembles the worst and most vitiated forms of syphilitic eruption, such for example as the large elevated, conical and concentric scab.

Such is the horror that the Greeks have of this disease, that they abandon their dearest and best friends; and the unfortunate victim is frequently obliged to seek refuge among the beasts of the field, and in the recesses of the mountains. An instance of this I saw in the plains of Argos, of a wretched man, affected with this loathsome malady, shunned by every one, friendless and homeless. Even when it appears among the better part of these people, it is considered a sufficient ground in either sex for a divorce, and it is sanctioned both by the civil and ecclesiastical laws.

Lepra is no longer considered as an incurable disease. It is more intractable and obstinate than common forms or cases of lues venerea, but is found to yield to proper treatment. The remedies most successful, are the same as in syphilis—mercurial and arsenical. Of all the forms of mercury, the corrosive sublimate, with sarsaparilla, is found the most efficacious. In obstinate cases, after the mercurial treatment has been long continued, without curative effects, the arsenical is substituted with the happiest results. The analogy here, too, is very striking, as every one must have observed the same thing in the treatment of the venereal disease."

4. *Facility of Union by the First Intention in Egypt.*—" The Egyptians are a very temperate people from necessity; there is no wine or ardent spirits peculiar to the country. To this, more than to climate alone, we would ascribe the greater readiness with which their diseases yield to treatment. From the state of nature in which they live, there is very little predisposition to inflammation; and hence the readiness with which they recover from wounds, and the remarkable success of surgical operations.

The salutary and desirable process of union by the first intention, or adhesion, is much more common and complete than in any part of Europe, or even in America. This has been ascribed by some to the heat and dryness of the climate alone; but we would give a part of the credit to the sound and natural constitutions of the Arabs. In the more civilized and refined countries of Europe and America, there is frequently either too much inflammation, or too high a degree of irritability, to have this object accomplished. Both these states of the system are well known by every surgeon to interfere with, and indeed frequently to frustrate, this process entirely.

Even the wound made in the operation of lithotomy, which is performed in the lateral way, except that the prostate and neck of the bladder are cut directly downwards towards the rectum as recommended and practised by Vacca, frequently heals by the first intention, as I was informed by Dr. Pruner, a very intelligent and distinguished German surgeon, at Cairo, who ranks high in the confidence of Mahomet Ali.

My experience in New York warrants me in saying, that the adhesive inflammation is, *cæteris paribus*, more favourable for union by the first intention, during our hot seasons, than in the cold weather of winter. This I have noticed in an abundance of instances, and have been in the habit of ascribing it to the lesser degree of inflammation that follows operations and injuries in the summer months."

*Aneurisms* are almost unknown in Egypt.

5. *The Plague.*—"Since visiting a number of oriental cities, it is no longer surprising to me that they should, from time to time, be scourged with typhoid forms of disease, and particularly the appalling and terrific forms of it, denominated the peste or plague. As long as their cities remain, and their habits continue, it must be, from time to time, the companion of the Mussulman. The features and appearance of this disease, like the Asiatic cholera, are frightful indeed, from the overwhelming operations of the contagion, infection or poison that produces it upon the nervous system. It certainly resembles the effect which some of the more deadly vegetable and animal poisons produce upon animal life. From the mild vegetable miasm that produces intermittent and remittent fevers, there is a variety of causes, vegetable and animal, differing in intensity and violence, until we arrive at the most concentrated of all, which is the *materies morbi* of plague itself.

From the facts which I collected at Cairo, Alexandria, Smyrna and Constantinople, in each of which places the disease existed, and in the first of which I saw a number of cases, my belief is, that it is not contagious, but infectious and atmospheric."

6. *Plague-clothes sent to London!* What will the quarantine people say to this? Conceive the horror of the Cockneys if they had suspected what a trick was being played on them.

"Doctor Pruner of Cairo informed me, that he never knew an instance of plague to follow an autopsy, among the pupils of the hospital, and that they made post-mortem examinations of plague subjects, as freely as those who die from other diseases. Dr. ——— of Alexandria, stated to us, that he sent the clothes and mattrass, of a person who had died of plague, to London, and that a quantity of the discharge from the charbons and buboes was mingled with them, and cotton was imbued with it purposely. It arrived safe, was taken home, but no disease communicated by it. His confidence in the non-contagiousness of plague was so great, that he was induced to make this bold and unjustifiable experiment."

We trust that Dr. Mott has regained the health he left the New World to seek.

### Dislocation of the Patella outwards, with Torsion on its Axis. By John Watson, M.D.

The following case is contained in our esteemed contemporary, the New York Journal of Medicine and Surgery, for October, 1839.

*Case.*—Henry Burton, a carman, of rather slender frame, and about thirty-five years of age, was on horseback in a crowd at the Park, August 21st, 1839, waiting to see the public reception of the Hon. Henry Clay. In the confusion of the crowd, another horseman backed his horse against him, and the animal's hip striking against Burton's right leg, injured him so severely that he was unable to say how, or in what direction he received the shock. He was carried immediately to a neighbouring hotel.

"I saw him soon after the accident, in connection with Dr. L. C. Ferris, Dr. Stearns of the northern part of the city, and Dr. Thomas F. Cock. The patient was complaining of severe pain; the leg was perfectly straight, but could be flexed about to an angle of 140 without increasing his suffering. The patella appeared to be slightly drawn up, and it was twisted upon its axis, presenting its outer edge, in a prominent hard line, in front of the knee: its inner edge was resting either in the groove between the condyles of the femur, upon which its posterior face should naturally play, or in the small indenture on the anterior face of the femur immediately above this groove. The anterior surface of the patella was turned inward, its posterior surface outward, and it rested nearly at right angles with its natural position. Its upper and lower attachments were both preserved, and could be distinctly felt; and a sort of band appeared to pass from its under, or, as it now lay, its outer face, inward to the deeper portion of the knee-joint. This band, as I conceived, was caused, either by the tension of the capsular ligament, or by the rupture of its edge, as it passes from the outer side of the patella. The position of the bone was so well marked that no one at all acquainted with the anatomy of the part, could mistake the nature of the accident."

With the leg extended, and the anterior muscles of the thigh forced downwards as much as possible, pressure was made upon the patella with the expectation of forcing down its prominent edge, and pushing the bone directly into its proper position. The effort was followed only by a severe increase of pain; the bone remained permanently fixed. Another attempt was made to cant its posterior edge inward, and to bring its anterior edge outward, without pressing it against the condyles of the femur, by forcing the head of a key against the posterior, now the outer face of the patella, (using this as a fulcrum,) and pressing the prominent edge of the bone towards the outer condyle. This manœuvre gave him no pain; but was as fruitless in its result as the other. At length the knee was forcibly bent, and immediately straightened again; and then, by canting the patella as before, and pushing it slightly downwards and inwards, it sprung, with a sudden snap, into its proper position. The reduction would, doubtless, have been facilitated by flexing the thigh strongly upon the pelvis. This, however, was not attempted, and the manipulation just described, answered every purpose.

As soon as the bone was replaced the pain ceased; and the patient thinking himself well again, stated that he could now walk as well as ever. A straight splint was applied behind the limb, so as to secure the joint, and the patient was sent home in a carriage, with directions to keep the knee at rest for a few days, and to apply to it an evaporating lotion.

After some observations on the imperfect notice of this accident, contained in works on surgery, Dr. Watson adds:—

"Though the position of the patella in this case was very different from that which it occupies in the subluxation outwards, yet it is easy to see how the latter accident might be resolved into this. For the patella in slipping outward, while passing over the outer edge of the pulley of the femur, must, from the very shape of the bones, have its outer edge tilted forward, and its inner edge, in consequence, thrown a trifle backward. Now, whilst in this position, and before the patella is allowed to reach fairly to the anterior face of the condyle, a sudden contraction of the rectus and vasti muscles must tend to bring the bone back again within the line of their action : so that instead of slipping completely over the condyle, it will either fall back into its natural position ; or its inner edge being arrested, and the bone being thus prevented from moving inwards bodily, its outer edge must be drawn forwards towards the axis of the limb, and the accident I have described will be thus produced."

This case adds another to the list of those in which flexion of the knee contributed towards the reduction.

---

### Mr. Donovan on a new Chemical Combination of Arsenic, Mercury, and Iodine ; and on its Medicinal Employment.*

"Triturate," says Mr. Donovan, " 6.08 grains of finely levigated metallic arsenic, 15.38 grains of mercury, and 50 grains of iodine, with one drachm measure of alcohol, until the mass has become dry, and from being deep brown has become pale-red. Pour on eight ounces of distilled water ; and after trituration for a few moments, transfer the whole to a flask ; add half a drachm of hydriodic acid, prepared by the acidification of two grains of iodine, and boil for a few moments. When the solution is cold, if there be any deficiency of the original eight ounces, make it up exactly to that measure with distilled water. Finally filter.

The theory of this process need scarcely be adverted to. By the long-continued trituration of arsenic, mercury, iodine, and alcohol, the metals are converted into iodides, which combine. The mass by solution in water is converted into an hydriodate of arsenic and mercury. The quantities of the two metals are so adjusted, that, when converted into protoxides by decomposition of a portion of the water in which they are dissolved, there will be eight grains of protoxide of arsenic, and sixteen of protoxide of mercury. The quantity of water is such that each drachm measure of the solution will contain exactly one-eighth of a grain of protoxide of arsenic, and one-fourth of a grain of protoxide of mercury. I conceive that the quantity of mercury ought to be double that of the arsenic, in order to insure a slow and moderate, yet adequate mercurial action, along with the proper effect of the arsenic.

Of this *liquor hydriodatis arsenici et hydrargyri*, each drachm measure consists of—

Water, one drachm.
Protoxide of arsenic, one-eighth of a grain.
Protoxide of mercury, one-fourth of a grain.
Iodine, (converted into hydriodic acid,) four-fifths of a grain.

The colour of the solution is yellow, with a pale tinge of green : its taste is slightly styptic. It cannot be properly conjoined with tincture of opium, or with sulphate, muriate, or acetate of morphia ; for all these produce immediate and copious precipitates in it. Hence if opiates are to be used during the exhibition of this arsenico-mercurial liquor, they must be taken at different

---

* Dub. Journ. Nov. 1839.

periods of the day. Tincture of ginger produces no bad effect. The following formula is proper:—

℞. Liquoris Hydriodatis Arsenici et Hydrargyri drachmas duas.
Aquæ Distillatæ uncias tres cum semisse.
Syrupi Zinziberis semunciam. Misce.
Divide in haustus quatuor. Sumatur unus mane nocteque.

Thus one-sixteenth of a grain of protoxide of arsenic, and one-fourth of a grain of protoxide of mercury, would be taken in each dose, along with two-fifths of a grain of iodine, which being in the state of combined hydriodic acid, will be much diminished in energy of medical effect. This is no doubt the proper dose to begin the exhibition of arsenic with; but it will be very soon necessary to increase it.

The division into draughts is here necessary: first, to insure accuracy of the dose, so essential in the case of this active medicine: and next, to prevent injury to the ingredients by the use of a metallic spoon as a measure—the general way in which, unfortunately, the dose of a medicine is determined."

Mr. Donovan recommends this in certain cases in which its ingredients have been found serviceable, and it deserves a trial.

---

### CASE OF ANEURYSM, CURED BY THE NEEDLE.

This case is quoted by our valued contemporary, the Medical Examiner, of Philadelphia,* from the Southern Medical and Surgical Journal, for July. It occurred to Mr. C. P. Richardson, of Savannah, Georgia.

*Case.*—"A. B., an Irish labourer, called at my office on the 27th of January last, with a tumor the size of a pigeon's egg, two inches above the wrist; the arm was much swollen and tense; its temperature greatly elevated, and, as may be supposed, he was labouring under great constitutional disturbance. The tumor was aneurismal; produced by a wound of the radial artery, an inch below the aneurism. The history which he gave me of the case was, that in some fight, three weeks before, he had been stabbed. At the time the wound bled considerably, but was stopped without surgical aid, and as the wound readily healed, no more was thought about it. In about a week afterwards, his arm became swollen from the elbow to the wrist, and very painful. The tumour then became obvious at the place I have mentioned. Its gradual increase, with the pain and tumefaction of his arm, induced him, two weeks afterwards, to seek advice. When I first saw him, his arm was much swollen with the blood that had infiltered throughout its whole cellular tissue; the heat and redness intense; his whole system participating in the irritation; and the aneurism tense and pulsating. It was at this time that I passed the needle horizontally through the tumour—using the precaution to arm the extremities of the needle with pieces of cork, that it might not escape or be caught by the roller with which I encircled the arm. Directions were then given to keep the arm constantly flexed—the bandage to be saturated with cold water—to take a large dose of salts, and to refrain from food. Previous to the passing of the needle, the pulsation of the tumour and the radial artery below the tumour, was distinctly felt. But shortly afterwards (ten minutes) the diastole of the artery was barely perceptible, whilst the pulsating of the tumour had altogether ceased. On the 30th,

* July 20, 1839.

three days after the first dressing, the tumour was hard, and no impression could be made by pressure—the pulsation of the artery below the tumour was very plain—the pain, heat, and tumefaction of the arm had subsided, with no appearance of suppuration at the point at which the needle was introduced."

In a week, the coagula sloughed out, and the artery above the tumor, as far as could be ascertained, was obliterated. Two weeks perfected the cure, and the man was able to go to work. Had the needle been withdrawn three days after its introduction, or before any signs of suppuration had made their appearance, Mr. R. believes that no sloughing would have occurred.

If we are to judge by this case, the method must be hazardous.

---

### Sloughing of a Brachial Aneurysm.*

Mr. Mackie, a surgeon of Cupar Fife, relates this case in our northern contemporary.

*Case.*—" Mr. W. G. of East Aytoun, near Scarborough, was bled in the median basilic about the 20th October, 1835, and unfortunately the point of the lancet penetrated the brachial artery. From the blood flowing *per saltum* it was apprehended that such was the case, and, in order to effect a cure, compression was employed, but without any effect, as in three weeks, a pulsating tumour, of the size of a hen's egg, had formed at the elbow, bearing the usual characteristics of aneurism.

In the course of another fortnight the surface of the swelling began to ulcerate, and in a week further, hemorrhage occasionally occurred, which so weakened and alarmed the patient, that he at last consented to submit to the operation of tying the brachial artery above the tumour, which, accordingly, was performed by me, in presence of Mr. Smart and Dr. Alexander, of Scarborough.

The operation was performed in the usual manner, by cutting down upon the artery along the inner margin of the biceps muscle. On tightening the ligature, the tumour almost instantly became flaccid, and pulsation at the wrist entirely ceased. The whole arm was then enveloped in new flannel, and an opiate exhibited, which soon had the effect of inducing tranquil sleep.

Mr. G. complained at first of an uneasy prickly feeling in the affected member, and the arm was rather cooler than the other. Sensation was in nowise affected. After reaction had commenced there was a good deal both of constitutional and of local irritation. The affected limb then became much hotter than the sound one, and general throbbing in the whole arm was then felt, synchronous with the pulsations of the heart, and, in about ten days, the pulse was indistinctly felt at the wrist. About this time also the whole aneurismal tumour sloughed away, leaving underneath a clear healthy surface, which rapidly healed. In a fortnight the ligature came away with the dressing, and in three weeks from the date of the operation, the patient had entirely recovered.

Three years afterwards, I had an opportunity of examining the arm, which was equally strong with the other, and the pulsations of either radial artery quite distinct."

---

* Edinburgh Med. and Surg. Journ. July, 1839.

